# Supplementary material for: Dietary Counseling Aimed at Reducing Sugar Intake Yields the Greatest Improvement in Management of Weight and Metabolic Dysfunction in Children with Obesity
Source: Nutrients. 2022 Apr 3;14(7):1500. doi: 10.3390/nu14071500 (PMC9003198; doi:10.3390/nu14071500)
Supplement: Supplementary file 1 [file nutrients-14-01500-s001.zip › nutrients-1655642-supplementary.pdf]

Supplemental Table S1. Health Behavior Score

Score/ Behavior	1	2	3	4
Second helpings	<1x per week	<2x per week	2-3x per week	>3x per week
Portion size	Age-appropriate	Occasional	Medium	Large
Sugar-sweetened beverages	<1x per month	<8 oz per day	<16 oz per day	>16 oz per day
Processed foods	<1x per month	≤1x per week	2-3x per week	>3x per week
Sweets	<1x per month	≤1x per week	2-3x per week	>3x per week
Unhealthy snacks	<1x per month	≤1x per week	2-3x per week	>3x per week
Skipping meals	<1x per month	≤1x per week	2-3x per week	>3x per week
Meals at school	<1x per month	≤1x per week	2-3x per week	>3x per week
Condiment use	<1x per month	Sometimes	Often	Regularly
Fruits and vegetables	≥5 servings/day	3-4 servings/day	1-2 servings/day	0-1 servings/day
Whole grains	≥4 servings/day	3 servings/day	2 servings/day	0-1 servings/day

Supplemental table S2: Baseline characteristics

Variable		Total	BMI-NR	BMI-R	p-value
Gender **No. (%)**	Female	71 (43.0%)	44 (62.0%)	27 (38.0%)	0.21
	Male	94 (57.0%)	49 (52.1%)	45 (47.9%)	
Race **No.(%)**	White	74 (44.8%)	39 (52.7%)	35 (47.3%)	0.71
	Black	47 (28.5%)	26 (55.3%)	21 (44.7%)	
	Hispanic	39 (23.6%)	25 (64.1%)	14 (35.9%)	
	Biracial	5 (3.0%)	3 (60.0%)	2 (40.0%)	
Obese mother BMI >30 No./total(%)		62/99 (62.6%)	43/62 (69.3%)	19/62 (30.6%)	**0.021**
Obese father BMI >30 No./total(%)		54/85 (63.5%)	32/54 (59.2%)	22/54 (40.7%)	0.691
Both parents overweight or obese No./total(%)		56/78 (71.8%)	35/56 (62.5%)	21/56 (37.5%)	0.518

Data are presented as mean (percent).
BMI = body mass index, NR = non-responders, R = responders
No. = number of patients
